# Interaction between the oxidative balance score and serum per- and poly-fluoroalkyl substances (PFASs) on liver health: analysis of the NHANES 2007–2018 dataset

**DOI:** 10.1265/ehpm.24-00159

**Published:** 2024-10-01

**Authors:** Ren Zhou, Fei Chen, Lei Zhang, Yu Sun, Hong Jiang, Rong Hu, Jia Yan

**Affiliations:** Department of Anesthesiology, The Ninth People’s Hospital of Shanghai, Jiao Tong University School of Medicine, Shanghai, 200011, PR China

**Keywords:** Liver markers, Per- and poly-fluoroalkyl substances, Oxidative balance score, Linear regression models, Bioaccumulation, Aspartate aminotransferase

## Abstract

**Background:**

Per- and poly-fluoroalkyl substances (PFASs) are pervasive synthetic compounds, prompting investigations into their intricate interactions with lifestyle factors and health indicators because of their enduring environmental presence and bioaccumulation. This study aimed to explore the effects of the oxidative balance score (OBS) and PFAS on liver-related indices.

**Methods:**

Twenty dietary and lifestyle factors were used to calculate the OBS. The serum concentrations of PFASs were measured, and their sum was calculated for analysis. The levels of liver markers were also evaluated. Linear regression models and interaction analyses were used to assess the associations between OBS, PFAS concentrations, and liver indices.

**Results:**

The results revealed an inverse association between high OBS and perfluorooctane sulfonic acid concentration, as well as the sum of PFAS concentrations. OBS was positively associated with liver markers. The PFAS concentrations were positively associated with total bilirubin, alanine aminotransferase (ALT), and aspartate aminotransferase (AST) levels. Interaction analyses revealed significant interactions between OBS and specific PFASs for alkaline phosphatase (interaction P < 0.05). Possible interactions were also found between OBS and specific PFASs for ALT, and AST levels (interaction P < 0.10).

**Conclusions:**

This study clarified the association between total PFAS and OBS. This association was significant mainly for diet-related OBS. PFAS and OBS are associated with liver-related indicators in the blood.

**Supplementary information:**

The online version contains supplementary material available at https://doi.org/10.1265/ehpm.24-00159.

## 1. Background

Per- and poly-fluoroalkyl substances (PFASs) constitute a group of synthetically produced chemicals that are extensively used in both industrial and consumer goods owing to their resistance to heat, water, and oil [1]. These chemicals are characterized by their unique chemical structure, which includes carbon-fluorine bonds, making them highly resistant to heat, water, and oil. Their persistent nature in the environment, along with their ability to bioaccumulate in living organisms, has raised concerns about their potential adverse effects on human health and the environment [2, 3].

Recently, concerns regarding the health implications of exposure to PFASs have increased. Epidemiological investigations have revealed an association between exposure to PFASs and various adverse outcomes. Analyses of the National Health and Nutrition Examination Survey (NHANES) data have specifically identified links between distinct PFASs and diverse adverse outcomes, including mortality [4], asthma [5], infertility [6], overweight/obesity [7], and common cold [8]. A comprehensive examination of cross-sectional studies conducted in a recent systematic review established correlations between several PFASs and increased levels of liver injury markers, including alanine aminotransferase (ALT), aspartate aminotransferase (AST), and gamma-glutamyl transferase (GGT) [9]. Alkaline phosphatase (ALP) and total bilirubin (TB), which are recognized indicators of liver damage, play pivotal roles in the clinical assessment of liver function and disease diagnosis.

The oxidative balance score (OBS), a metric for evaluating an individual’s overall antioxidant status [10], combines antioxidant and prooxidant components from dietary and lifestyle factors. A high OBS indicates greater antioxidant exposure relative to prooxidant exposure. Studies have revealed an association between OBS and blood or urinary oxidative stress markers. One study revealed that a low OBS is associated with biological aging [11], calculated from various blood indicators, including oxidative stress-related indicators. This reflects the ability of OBS to indicate overall health. A Korean population-based study revealed an association between OBS and GGT [12], and a Japanese population-based study revealed an association between OBS and urinary 8-hydroxydeoxyguanosine [13]. These studies demonstrate a direct link between OBS and the level of oxidative stress in the body. Studies have also highlighted its association with various health conditions, including cardiovascular [14] and chronic kidney diseases [15], colorectal adenomas [16], cancer, and mortality [17].

Dietary and lifestyle factors influence human exposure to PFASs. Our recent investigations revealed associations between dietary inflammatory indices and blood levels of multiple PFASs [18]. Furthermore, habits such as alcohol consumption and smoking are additional factors impacting exposure [19] to PFASs. The impact of PFASs on human health is more pronounced in populations with specific lifestyle habits or dietary patterns [20]. However, previous studies are limited by their examination of a single factor, and, in recognition of the complexity of human health, a comprehensive metric such as OBS may be more effective in evaluating the overall impact of the adverse effects of PFASs on human health.

Therefore, this study aimed to explore the association between OBS and PFAS serum concentrations based on the NHANES dataset. The effects of OBS and PFASs on liver-related indices were assessed, and possible interactions were evaluated.

## 2. Methods

### 2.1. Study population

The NHANES, conducted by the National Center for Health Statistics (NCHS) under the Centers for Disease Control and Prevention (CDC), has employed a complex sampling framework to represent the US population since 1960. This survey’s protocol was approved by the NCHS Research Ethics Review Board, with all participants providing informed consent. For the analysis, we used 12 years of NHANES data (2007–2018), which can be freely accessed from the CDC’s official website and combined according to the NHANES tutorial guidelines (https://wwwn.cdc.gov/nchs/nhanes/tutorials/default.aspx).

**Fig. 1:**
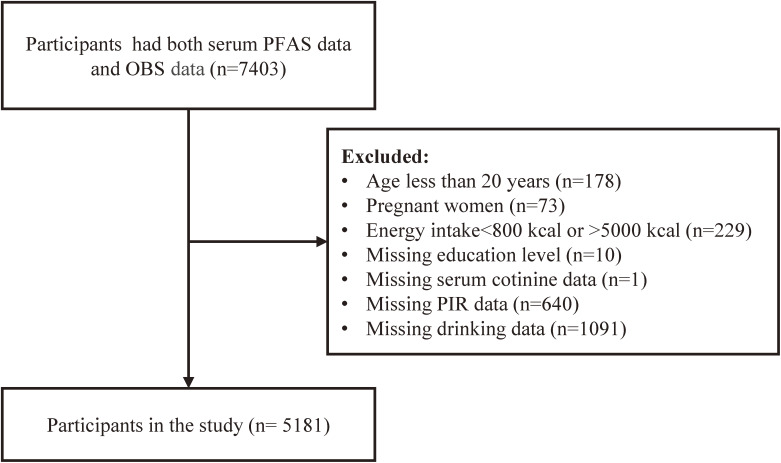
Schematic flowchart illustrating participant selection and exclusion criteria

Among the participants, 7,403 had data for both OBS calculations and PFASs. Of these, 178 participants aged <20 years, 73 pregnant women, and 229 participants with an average daily caloric intake ⩽800 kcal or ⩾5000 kcal were excluded from the study. After excluding individuals with missing covariate data, 5,181 participants were included in the analyses. The flow chart of participant selection is shown in Fig. 1.

### 2.2. Assessment of OBS

The OBS was computed by evaluating 16 nutrients and four lifestyle elements, encompassing five prooxidants and 15 antioxidants [21]. This assessment relied on a priori knowledge of the association between oxidative stress and specific nutrients or lifestyle factors.

For nutrient data, we combined two 24-h dietary recalls from the NHANES database. Two 24-h dietary recalls were collected using the US Department of Agriculture’s automated multiple-pass method [22]; the first 24-h recall was administered in person in the Mobile Examination Center (MEC), and the second was completed via telephone 3–10 days later. Participants were given measuring cups, spoons, a ruler, and a food model booklet, which contained two-dimensional drawings of the various measuring guides available in the MEC, to use for reporting food amounts during the telephone interview. Details on the dietary recall method can be found on the NHANES website (https://www.cdc.gov/nchs/nhanes/measuring_guides_dri/measuringguides.htm).

Prooxidant dietary factors included saturated fatty acids (SFAs), omega-6 polyunsaturated fatty acids (PUFAs), total iron, smoking status, drinking status, and obesity status. SFAs, omega-6 PUFAs, and total iron intake were assigned scores ranging from 0 to 2 points corresponding to the sex-specific tertile values categorized into low (2), intermediate (1), and high (0) levels. The antioxidant dietary factors included omega-3 PUFAs, vitamin C, vitamin E, selenium, total beta-carotene, fiber, retinol, monounsaturated fatty acids (MUFAs), and zinc. Points ranging from 0 to 2 were assigned based on the sex-specific tertile values for each variable and categorized into high (2), intermediate (1), and low (0) levels.

Lifestyle factors, including physical activity, body mass index (BMI), alcohol consumption, and smoking, were also considered. Based on the level of alcohol consumption, participants were categorized into three groups: heavy drinkers (⩾15 g/d for women and ⩾30 g/d for men), nonheavy drinkers (0–15 g/d for women and 0–30 g/d for men), and nondrinkers, with 0, 1, and 2 points, respectively.

Smoking status was scored as “2” for never smokers, “1” for former smokers, and “0” for current smokers. Obesity status was assessed as “2” for normal weight (BMI <25), “1” for overweight (25 ⩽ BMI < 30), and “0” for obesity (BMI ⩾30). Participants’ physical activity was scored from “0” to “2” based on the tertile values of the metabolic equivalent of task (MET) score and categorized into high (2), intermediate (1), and low (0) groups. The total OBS, determined by summing the points assigned to each factor, ranged from 0 to 40. The dietary OBS was the sum of the 16 dietary factors, while the lifestyle OBS was the sum of the four lifestyle factors.

For analysis, the OBS data were categorized into weight tertiles: tertile 1 (0–33.3%), tertile 2 (33.3–66.6%), and tertile 3 (66.6–100%). Due to the narrow range of values, the three classifications of lifestyle OBS were appropriately adjusted to ensure balance.

### 2.3. PFAS measurement

In this study, five PFASs—perfluorononanoic acid (PFNA), perfluorooctanoic acid (PFOA), perfluorodecanoic acid (PFDA), perfluorooctane sulfonic acid (PFOS), and perfluorohexane sulfonic acid (PFHxS)—with each exhibiting a detection rate of more than 80% were selected. Over the survey cycles, from 2013 to 2018, the NHANES separately measured the linear and branched isomers of PFOA and PFOS and combined their values as the serum concentrations.

The detection and quantification of serum PFAS biomarkers employed solid-phase extraction with high-performance liquid chromatography–turbo ion spray ionization tandem mass spectrometry, as detailed on the NHANES website (https://wwwn.cdc.gov/Nchs/Nhanes/2015-2016/PFAS_I.htm). The total concentration of PFASs (sum-PFASs), derived from the sum of the concentrations of the five specified PFASs, was also used for analysis. When the serum PFAS biomarker levels were below the limit of detection (LOD), we assigned a value equal to the LOD divided by the square root of 2. Both individual and sum-PFAS concentrations were log-transformed during analysis. In some analyses, the PFAS were categorized into weight tertiles: tertile 1 (0–33.3%), tertile 2 (33.3–66.6%), and tertile 3 (66.6–100%).

### 2.4. Oxidative stress and inflammatory marker measurements

Five blood markers associated with liver function were selected for our study: TB, ALP, GGT, ALT, and AST. The methods used were mostly consistent. Slight variations in the analyzers were observed across different cycles. A Beckman Coulter LX20 analyzer (2007–2008), Beckman UniCel® DxC800 analyzer (2007–2016), and Roche Cobas 6000 (c501 module) analyzer (2017–2018) were used for the measurements. The analysis methods are described in detail on the NHANES website (https://www.cdc.gov/nchs/nhanes/index.htm).

### 2.5. Covariates

The model incorporated the following variables as covariates: age (a continuous variable), sex, education (categorized as completed less than high school, completed high school, some college or Associate of Arts [AA] degree, and college graduate or above), race (classified as non-Hispanic White, non-Hispanic Black, Hispanic [Mexican-American and other Hispanic], and other), poverty income ratio (PIR, a continuous variable), BMI (a continuous variable), smoking status (categorized as never smoked, former smoker, and current smoker), drinking status (categorized as no alcohol use, 1–4 drinks per week, and >4 drinks per week), total energy intake (a continuous variable), physical activity (measured in MET per week, a continuous variable), history of liver disease, and serum cotinine levels for evaluating smoking status (a continuous variable).

History of liver disease was obtained by asking participants: “Has a doctor or other health professional ever told you that you had any kind of liver condition?”

The MET per week, indicating the duration of physical activity during a typical week, was computed according to the guidelines of the Global Physical Activity Questionnaire. Total daily energy intake was derived from dietary interview data.

### 2.6. Statistical analysis

The CDC guidelines were followed for all the statistical analyses, which included calculating estimates while accounting for the NHANES sample weights. Participant demographics were analyzed using descriptive statistics. Analyses of variance and chi-square tests were used to analyze the differences in characteristics across OBSs.

Weight linear regression models were used to examine the association between the OBS and PFAS concentrations and to analyze the impact of OBS and PFAS on liver function markers. The effects of PFASs on liver markers in populations with different OBSs were assessed using linear regression. The linear regression results are presented as regression coefficients (β), 95% confidence intervals (CIs) for β, and P-values. Significance was set at a P-value <0.05. P-values are shown as specific values, with P values less than 0.001 indicated as <0.001. We performed tests for linear trend by entering the median value of each category of OBS as a continuous variable in the models. The P-value for the interaction was obtained by using analysis of variance to compare fitting the regression equation with the interaction term and fitting the regression equation without the interaction term.

## 3. Results

### 3.1. Baseline characteristics

The baseline characteristics of the study participants are presented in Table 1. The mean age of the participants was 50.41 years; and 58.9% were men. Most participants were non-Hispanic white. The mean OBS and 95% CI was 22.52 [22.33–22.71]. The mean and 95%CI values for dietary and lifestyle OBS were 17.64 [17.46–17.82] and 4.88 [4.84–4.92], respectively (Table 1).

**Table 1:** Weighted baseline characteristics of the participants

	**ALL**	**Tertile 3**	**Tertile 2**		**Tertile 1**		**Overall** **P value**
			**Value**	**P value compared T3**	**Value**	**P value compared T3**	
**Age (years), mean [95% CI]**	50.41 [49.94–50.89]	48.28 [47.42–49.14]	50.24 [49.42–51.06]	0.002	52.18 [51.39–52.96]	<0.001	<0.001
**Sex**				0.82		>0.999	0.954
Male, %	48.9	51.7	49.5		45.7		
Female, %	51.1	48.3	50.5		54.3		
**Poverty income ratio**				<0.001		<0.001	<0.001
<1.3, %	10.9	8.2	9.8		14.6		
1.3–3.5, %	64.1	60.4	67.2		64.7		
>3.5, %	25	31.4	23.0		20.7		
**BMI (kg/m2), mean**				<0.001		<0.001	<0.001
<25, %	40	32.9	40.9		46.2		
25–29.9, %	31.7	33.5	29.6		32.1		
>29.9, %	28.2	33.6	29.5		21.7		
**Race**				0.147		<0.001	<0.001
Hispanic, %	13.1	13.1	14.1		12.2		
Non-Hispanic White, %	70.2	73.1	70.1		67.4		
Non-Hispanic Black, %	9.6	6.6	8.2		14.1		
Other Race, %	7.1	7.3	7.7		6.2		
**Education level**				<0.001		<0.001	<0.001
Less than high school, %	13.2	8.1	11.9		19.3		
High school graduate/GED or equivalent, %	23.5	18.5	24.3		27.4		
Some college or AA degree, %	31.4	31.1	29.7		33.5		
College graduate or above, %	31.9	42.2	34.1		19.8		
**Smoking status**				<0.001		<0.001	<0.001
Current, %	18.5	9.1	15.9		30.3		
Never, %	24.8	23.3	26.4		24.6		
Former, %	56.7	67.6	57.7		45.1		
**Alcohol consumption**				>0.999		<0.001	0.616
Never	18.2	16.9	17.4		20.2		
not more than 4 drinks per week, %	77.2	78.2	77.9		75.5		
more than 4 drinks per week, %	4.6	4.9	4.7		4.2		
**Serum cotinine (ng/mL), mean ** **[95% CI]**	57.1 [53.57–60.64]	29.02 [24.33–33.72]	49.81 [43.73–55.89]		84.69 [78.14–91.25]	<0.001	<0.001
**PFOA detected rate, %**	99.6	100.0	99.5	0.073	99.3	0.022	0.041
**Blood PFOA concentrations ** **(ng/mL), mean [95% CI]**	2.91 [2.83–2.98]	2.95 [2.8–3.1]	2.85 [2.75–2.95]	0.704	2.93 [2.79–3.06]	0.467	0.762
**PFNA detected rate, %**	98.2	98.0	98.5	0.658	98.1	0.929	0.845
**Blood PFNA concentrations ** **(ng/mL), mean [95% CI]**	1.09 [1.06–1.13]	1.06 [1.01–1.11]	1.09 [1.04–1.13]	0.215	1.13 [1.07–1.19]	0.475	0.473
**PFOS detected rate, %**	99.7	100.0	99.9	0.073	99.3	0.022	0.041
**Blood PFOS concentrations ** **(ng/mL), mean [95% CI]**	11.23 [10.61–11.86]	10.75 [8.85–12.65]	10.78 [10.2–11.35]	0.02	12 [11.39–12.61]	<0.001	<0.001
**PFDA detected rate, %**	82.4	83.3	83.2	0.436	80.7	0.046	0.114
**Blood PFDA concentrations ** **(ng/mL), mean [95% CI]**	0.31 [0.3–0.33]	0.3 [0.29–0.32]	0.32 [0.29–0.34]	0.763	0.32 [0.3–0.34]	0.544	0.635
**PFHxS detected rate, %**	99.3	99.9	99.3	0.02	98.7	0.001	0.004
**Blood PFHxS concentrations ** **(ng/mL), mean [95% CI]**	2.15 [2.07–2.23]	2.15 [1.98–2.32]	2.05 [1.94–2.16]	0.795	2.23 [2.11–2.36]	0.275	0.31
**Sum-PFAS detected rate, %**	99.9	100.0	100.0	1	99.6	0.247	0.098
**Blood Sum-PFAS concentrations ** **(ng/mL), mean [95% CI]**	17.7 [17.01–18.39]	17.21 [15.23–19.2]	17.08 [16.36–17.81]	0.101	18.6 [17.82–19.39]	0.002	0.009
**OBS, mean [95% CI]**	22.52 [22.33–22.71]	30.67 [30.57–30.78]	24 [23.91–24.1]	<0.001	15.07 [14.9–15.23]	<0.001	<0.001
**Dietary OBS, mean [95% CI]**	17.64 [17.46–17.82]	25.17 [25.07–25.28]	19.15 [19.04–19.26]	<0.001	10.63 [10.47–10.79]	<0.001	<0.001
**Lifestyle OBS, mean [95% CI]**	4.88 [4.84–4.92]	5.5 [5.43–5.57]	4.85 [4.78–4.92]	<0.001	4.44 [4.37–4.5]	<0.001	<0.001
**Serum Total Bilirubin** **TB (mg/dL), mean [95% CI]**	0.65 [0.64–0.66]	0.67 [0.65–0.69]	0.66 [0.64–0.67]	0.479	0.63 [0.62–0.65]	<0.001	<0.001
**Alkaline phosphatase** **ALP (IU/L), mean [95% CI]**	70.46 [69.82–71.1]	67.39 [66.3–68.47]	70.63 [69.43–71.82]	<0.001	72.64 [71.59–73.68]	<0.001	<0.001
**Gamma glutamyl transferase ** **GGT (U/L), mean [95% CI]**	27.5 [26.58–28.42]	25.2 [23.86–26.54]	28.88 [26.79–30.96]	<0.001	28.05 [26.82–29.27]	<0.001	<0.001
**Aspartate aminotransferase ** **AST (U/L), mean [95% CI]**	24.37 [23.84–24.91]	24.89 [24.04–25.74]	25.09 [23.89–26.28]	0.219	23.36 [22.66–24.05]	<0.001	<0.001
**Aspartate aminotransferase ** **AST (U/L), mean [95% CI]**	24.64 [24.16–25.12]	25.25 [24.61–25.89]	24.99 [23.82–26.16]	0.007	23.87 [23.32–24.43]	<0.001	<0.001

Continuous variable data are presented as weighted medians and P25–P75, categorical data are presented as weighted proportions. For comparisons between the two groups, P < 0.025 was considered significant (bonferroni correction). Abbreviations: PFAS, perfluoroalkyl and polyfluoroalkyl substances; PFOA, perfluorooctanoic acid; PFNA, perfluorononanoic acid; PFOS, perfluorooctane sulfonic acid; PFDA, perfluorodecanoic acid; PFHxS, perfluorohexane sulfonic acid; OBS, oxidative balance score; BMI, body mass index; PIR, poverty income ratio.

Significant differences in the participants’ age, PIR, BMI, race, education, and smoking status were observed between the OBS tertiles. PFOA, PFOS, and PFHxS were detected at higher rates in the higher OBS group. PFOS and sum-PFAS were detected at lower concentrations in the higher OBS group (Table 1). Contingency table for OBS and PFAS tertiles were shown in Table S1.

### 3.2 Association between OBS and serum PFASs

OBS was significantly and inversely associated with serum PFOS and sum-PFAS concentrations. The β value was −0.008 (95%CI: −0.013, −0.002) for OBS and PFOS and −0.005 (95%CI: −0.009, 0.000) for sum-PFAS. Between OBS tertile 1 and tertile 3, the β values were 0.120 (95%CI: 0.032, 0.209) for PFOS and 0.07 (95%CI: −0.007, 0.147) for sum-PFAS. Further analysis revealed that dietary OBS was associated with both the PFOS and sum-PFAS scores. However, only one association was found between lifestyle OBS and serum PFHxS concentrations. The results are summarized in Table 2.

**Table 2:** Association between blood PFAS and OBS.

**Chemicals**	**OBS level**	**OBS**	**Dietary-OBS**	**Lifestyle-OBS**
		**β (95%CI)**	**β (95%CI)**	**β (95%CI)**
PFOA	High OBS	Reference	Reference	Reference
	Moderate OBS	−0.003 (−0.07, 0.065)	−0.011 (−0.087, 0.064)	−0.01 (−0.094, 0.075)
	Low OBS	0.027 (−0.044, 0.098)	0.058 (−0.02, 0.137)	−0.029 (−0.139, 0.08)
	Per 1 unit OBS increase*	−0.003 (−0.007, 0.002)	−0.003 (−0.008, 0.001)	0.01 (−0.016, 0.037)
	P trend**	0.396	0.083	0.571

PFNA	High OBS	Reference	Reference	Reference
	Moderate OBS	0.056 (−0.041, 0.154)	0.03 (−0.063, 0.124)	0.019 (−0.073, 0.111)
	Low OBS	0.065 (−0.043, 0.174)	0.078 (−0.03, 0.185)	0.08 (−0.048, 0.209)
	Per 1 unit OBS increase*	−0.005 (−0.011, 0.002)	−0.004 (−0.011, 0.003)	−0.011 (−0.044, 0.022)
	P trend**	0.266	0.143	0.193

PFDA	High OBS	Reference	Reference	Reference
	Moderate OBS	0.02 (−0.07, 0.11)	−0.008 (−0.098, 0.082)	0.069 (−0.019, 0.156)
	Low OBS	0.014 (−0.083, 0.112)	−0.004 (−0.102, 0.095)	0.13 (−0.009, 0.268)
	Per 1 unit OBS increase*	−0.001 (−0.006, 0.004)	−0.001 (−0.006, 0.005)	−0.015 (−0.049, 0.019)
	P trend**	0.805	0.962	0.074

PFOS	High OBS	Reference	Reference	Reference
	Moderate OBS	0.081 (−0.007, 0.168)	0.05 (−0.037, 0.138)	−0.031 (−0.122, 0.059)
	Low OBS	**0.12** **(0.032, 0.209)**	**0.122** **(0.032, 0.213)**	−0.067 (−0.182, 0.048)
	Per 1 unit OBS increase*	**−0.008** **(−0.013, −0.002)**	**−0.009** **(−0.015, −0.003)**	0.018 (−0.016, 0.051)
	P trend**	**0.010**	**0.006**	0.243

PFHxS	High OBS	Reference	Reference	Reference
	Moderate OBS	−0.03 (−0.111, 0.052)	−0.019 (−0.108, 0.07)	**−0.131** **(−0.244, −0.018)**
	Low OBS	−0.019 (−0.119, 0.082)	0.014 (−0.085, 0.112)	**−0.25** **(−0.404, −0.095)**
	Per 1 unit OBS increase*	0.004 (−0.003, 0.01)	0.003 (−0.004, 0.009)	0.042 (−0.001, 0.086)
	P trend**	0.778	0.696	**0.002**

Sum-PFAS	High OBS	Reference	Reference	Reference
	Moderate OBS	0.04 (−0.032, 0.112)	0.02 (−0.055, 0.094)	−0.035 (−0.117, 0.048)
	Low OBS	0.07 (−0.007, 0.147)	**0.082** **(0.003, 0.161)**	−0.072 (−0.175, 0.031)
	Per 1 unit OBS increase*	**−0.005** **(−0.009, 0.000)**	**−0.005** **(−0.01, −0.001)**	0.016 (−0.012, 0.045)
	P trend**	0.079	**0.030**	0.160

Model were adjusted for survey cycle, sex, age, race/ethnicity, education level, smoking status, BMI, serum cotinine, PIR, energy intake, physical activity, have any liver disease, and alcohol consumption.

*: Calculated by including the OBS score directly as a continuous variable in the models.

**: Calculated by entering the median value of each OBS category as a continuous variable in the models.

Abbreviations: PFAS, perfluoroalkyl and polyfluoroalkyl substances; PFOA, perfluorooctanoic acid; PFNA, perfluorononanoic acid; PFOS, perfluorooctane sulfonic acid; PFDA, perfluorodecanoic acid; PFHxS, perfluorohexane sulfonic acid; OBS, oxidative balance score; BMI, body mass index; PIR, poverty income ratio; CI, confidence interval.

### 3.3 Association between OBS and serum liver function markers

OBS was positively associated with serum ALT and AST levels and negatively associated with serum ALP levels. The increases in the β values of OBS and ALT, AST, and ALP were 0.005 (95%CI: 0.001, 0.008), 0.004 (95%CI: −0.009, 0.000), and −0.005 (95%CI: −0.007, −0.002), respectively. Further analysis revealed that dietary OBS had a similar association with total OBS. However, lifestyle OBS was only inversely associated with GGT (−0.038; 95%CI: −0.066, −0.01) (Table 3). After adjustment for total PFAS concentration, this association remained similar (Table S2).

**Table 3:** Association between OBS and liver function markers.

**Chemicals**	**OBS level**	**OBS**	**Dietary-OBS**	**Lifestyle-OBS**
		**β (95%CI)**	**β (95%CI)**	**β (95%CI)**
TB	High OBS	Reference	Reference	Reference
	Moderate OBS	−0.013 (−0.079, 0.052)	−0.001 (−0.062, 0.061)	−0.06 (−0.126, 0.006)
	Low OBS	−0.028 (−0.098, 0.041)	−0.011 (−0.088, 0.066)	−0.054 (−0.137, 0.028)
	Per 1 unit OBS increase*	0.002 (−0.003, 0.006)	0.002 (−0.003, 0.006)	−0.01 (−0.04, 0.02)
	P trend**	0.413	0.750	0.273

ALP	High OBS	Reference	Reference	Reference
	Moderate OBS	0.038 (0.000, 0.075)	0.019 (−0.017, 0.055)	0.012 (−0.026, 0.049)
	Low OBS	0.067 (0.022, 0.112)	**0.067** **(0.027, 0.106)**	−0.024 (−0.087, 0.039)
	Per 1 unit OBS increase*	**−0.005** **(−0.007, −0.002)**	**−0.005** **(−0.008, −0.003)**	0.009 (−0.007, 0.024)
	P trend**	**0.004**	**<0.001**	0.358

GGT	High OBS	Reference	Reference	Reference
	Moderate OBS	0.013 (−0.053, 0.078)	−0.003 (−0.064, 0.057)	**0.092** **(0.023, 0.16)**
	Low OBS	0.014 (−0.064, 0.092)	0.003 (−0.077, 0.082)	**0.177** **(0.054, 0.301)**
	Per 1 unit OBS increase*	−0.002 (−0.006, 0.003)	0.000 (−0.005, 0.005)	**−0.038** **(−0.066, −0.01)**
	P trend**	0.744	0.925	**0.007**

ALT	High OBS	Reference	Reference	Reference
	Moderate OBS	−0.025 (−0.074, 0.025)	−0.022 (−0.069, 0.025)	−0.007 (−0.065, 0.052)
	Low OBS	−0.058 (−0.125, 0.009)	−0.059 (−0.127, 0.01)	0.013 (−0.076, 0.101)
	Per 1 unit OBS increase*	**0.005** **(0.001, 0.008)**	**0.006** **(0.002, 0.009)**	−0.016 (−0.035, 0.004)
	P trend**	0.088	0.086	0.729

AST	High OBS	Reference	Reference	Reference
	Moderate OBS	−0.024 (−0.065, 0.017)	−0.015 (−0.051, 0.021)	−0.055 (−0.117, 0.007)
	Low OBS	−0.056 (−0.104, −0.008)	−0.05 (−0.094, −0.005)	−0.063 (−0.134, 0.008)
	Per 1 unit OBS increase*	**0.004** **(0.002, 0.007)**	**0.004** **(0.001, 0.007)**	0.01 (−0.003, 0.024)
	P trend**	**0.020**	**0.022**	0.098

Model were adjusted for survey cycle, sex, age, race/ethnicity, education level, smoking status, BMI, serum cotinine, PIR, energy intake, physical activity, have any liver disease, and alcohol consumption.

*: Calculated by including the OBS score directly as a continuous variable in the models.

**: Calculated by entering the median value of each OBS category as a continuous variable in the models.

Abbreviations: PFAS, perfluoroalkyl and polyfluoroalkyl substances; PFOA, perfluorooctanoic acid; PFNA, perfluorononanoic acid; PFOS, perfluorooctane sulfonic acid; PFDA, perfluorodecanoic acid; PFHxS, perfluorohexane sulfonic acid; OBS, oxidative balance score; BMI, body mass index; PIR, poverty income ratio; CI, confidence interval.

### 3.4 Association between PFASs and serum liver function markers

TB was positively associated with PFHxS (0.037; 95%CI: 0.015–0.060) and sum-PFAS (0.044; 95%CI: 0.010–0.078). Additionally, a nonsignificant trend for PFOA (0.043; 95%CI: −0.002–0.088), PFNA (0.034; 95%CI: −0.004 to 0.072), and PFOS (0.029; 95%CI: 0.000 to 0.057) was observed. ALT and AST also exhibited positive associations with PFOA, PFNA, and PFHxS, with ALT presenting β values for PFOA (0.033, 95%CI: 0.003 to 0.062, PFNA (0.031, 95%CI: 0.005 to 0.057), and PFHxS (0.033, 95%CI: 0.011 to 0.054). AST demonstrated β values for PFOA (0.028, 95%CI: 0.009 to 0.046) and PFHxS (0.026, 95%CI: 0.009 to 0.042). Fewer associations were noted between GGT and ALP with PFAS (Table S3).

### 3.5 Interaction between PFASs and OBSs on serum liver function markers

**Fig. 2:**
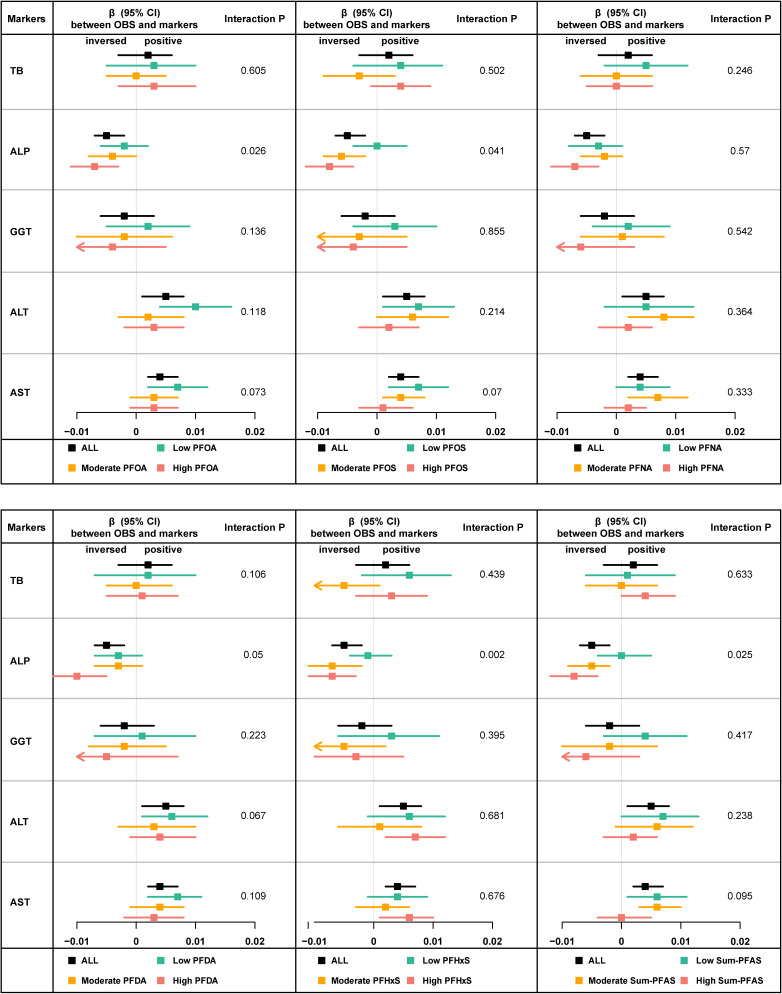
Forest plots show the relationship between OBS and TB, ALP, GGT, ALT, and AST at different PFAS levels. Forest plots display the regression coefficients (with 95% confidence intervals) between OBS (as continuous variables) and liver markers across three PFAS tertiles. These PFAS tertiles are defined as follows: Low PFAS (0–33%), Moderate PFAS (33%–66%), and High PFAS (66%–100%). Each square point on the plot represents a regression coefficient (β value), with error bars indicating the 95% confidence interval. The vertical line at 0 denotes no correlation. The linear regression was adjusted for the survey cycle, sex, age, race/ethnicity, education level, smoking status, BMI, serum cotinine, PIR, total energy intake, physical activity, history of liver disease, and alcohol consumption. Abbreviations: CI, confidence interval; PFAS, perfluoroalkyl and polyfluoroalkyl substance; PFOA, perfluorooctanoic acid; PFNA, perfluorononanoic acid; PFOS, perfluorooctane sulfonic acid, PFDA, perfluorodecanoic acid; PFHxS, perfluorohexanesulfonic acid; OBS, oxidative balance score; TB, total bilirubin; ALP, alkaline phosphatase; GGT, gamma-glutamyl transferase; AST, aspartate aminotransferase; ALT, alanine aminotransferase; BMI, body mass index; PIR, poverty income ratio.

Interaction analyses revealed significant interactions between OBS and specific PFASs for ALP. With the exception of PFNA, the associations of OBS with ALP levels were more significant in individuals with higher PFAS levels. PFOA, PFOS and total PFAS showed a possible interaction (P < 0.10) with OBS in the association with AST (Fig. 2). Dietary OBS showed similar associations to those observed for total OBS (Fig. S1). For lifestyle OBS, no possible interaction between PFAS and ALP was observed (Fig. S2).

## 4. Discussion

In this study, we observed an inverse association between OBS and the serum concentrations of PFOS and sum-PFASs in adults in the US. Dietary factors also influenced this association. Our study revealed that OBS was associated with a series of serum liver function markers. PFASs were also associated with several liver function markers. Furthermore, the association between OBS and several liver function markers was greater in individuals with special PFASs level. Dietary factors played a key role in this interaction.

An inverse association was observed between the OBS and blood concentrations of various PFASs. Furthermore, dissecting OBS into diet-related and lifestyle-related OBSs revealed that the inverse association was significant only in terms of diet-related OBS. The crucial role of diet in exposure to PFASs has been widely recognized. Dietary fiber [23], fish products [24], and other dietary factors have been reported to influence PFAS concentrations. Our previous study revealed that the Dietary Inflammatory Index (DII), a score indicating the overall dietary inflammation level, was positively correlated with various blood PFAS concentrations [18]. The DII contains similar but more dietary factors than the OBS. This new finding demonstrates that even with fewer dietary indicators, the associations between dietary factors and some PFASs remain consistent. However, not all the PFAS, DII, and OBS data demonstrated consistent associations. While high DIIs were associated with higher PFNA blood levels, OBS did not reveal a significant association. In contrast, the effect of the OBS on the sum of the PFASs was more significant than that of the DII. Although the DII and OBS share many indicators, they still differ in their dietary status based on previous literature. In previous NHANES-based studies, more than half of the participants consumed only proinflammatory or prooxidant diets. These results highlight the importance of considering multiple aspects of dietary factors in future studies. Although lifestyle habits such as alcohol consumption and BMI have been associated with blood PFAS concentrations in the literature [19], our study did not find a significant relationship between lifestyle OBS and blood PFAS concentration, adding a nuanced perspective to the ongoing discourse in environmental health research. Our study underscores the pervasive influence of dietary and lifestyle choices on individual exposure to environmental pollutants and contributes novel insights to the existing body of research. This finding extends our understanding beyond direct environmental sources, emphasizing the need to consider the broader context of daily behaviors in comprehending PFAS bioaccumulation.

A positive correlation was observed between OBS and several liver function markers, such as ALT and AST. Previous studies based on NHANES data reported that poor diets, such as inflammatory diets, were associated with lower ALT and AST levels, similar to our study [25]. Moreover, a randomized controlled study reported that a high anti-inflammatory diet for a specific period lowered ALT and AST levels in participants [26]. Interestingly, OBS and ALP showed a negative association. In liver damage caused by many compounds, the changes in ALP, AST, and ALT tend to occur in the same direction. However, there are also a range of compounds that cause ALP to change in the opposite direction to AST and ALT [27, 28]. It is hypothesized that this may be because ALP is not mainly tissue specific. It also responds to changes in different system like skeletal systems [29], whereas AST and ALT are more focused on liver changes. This observation highlights the complexity of the influence of diet on liver health. Concurrently, PFASs were positively correlated with these liver indices, indicating the potential adverse impact of environmental exposure on liver health. This finding aligns with the established research on environmental health, showing the consistent nature of these associations across various studies. These findings indicate a potentially distinct mode of action of diet and PFAS on liver function, further emphasizing the complex interplay between dietary and lifestyle choices and environmental factors influencing liver function. This understanding is crucial for informing future research directions in environmental health and advancing our understanding of the intricate relationships in this field.

When we explored population stratification based on PFAS, a compelling interaction effect became apparent, and a similar interaction was also found between dietary OBS and PFAS. Previous animal studies have demonstrated that nutrient intake can mitigate the toxic effects of environmental pollutants [30]. Recent investigations have revealed that certain dietary habits can interact with the toxic effects of environmental pollutants in normal populations. For instance, an anti-inflammatory diet counteracts the effects of bisphenol A on mortality [31] and reduces the impact of PFAS on blood oxidative stress and inflammatory markers [18]. Our study further underscores the relationship between dietary habits and the health consequences of environmental pollutants. Given the challenges in avoiding exposure to environmental pollutants in the current social environment, dietary interventions have emerged as a potential protective strategy. Notably, prior studies have identified lifestyle habits such as smoking [32], alcohol consumption, or physical activity as environmental pollutants with adverse effects [33, 34]. One study reported an interaction between alcohol intake, PFAS, and liver markers [19]. Our study did not reveal a discernible trend in the interaction between OBS and PFAS lifestyle habits with respect to liver function indices. This lack of a trend may be attributed to the specific compounds studied or adverse outcomes observed, emphasizing the need for further investigation of the role of lifestyle habits in the adverse effects of environmental agents.

This study has several strengths. First, we analyzed data from the NHANES. We applied a tutorial-based weighting method to ensure that our results properly represented the overall population. Second, the large sample size helped us adjust for various factors, increasing the accuracy of our study. However, this study also had a few limitations. One main concern is that our dietary data were based on a 24-h review, which may not capture long-term dietary patterns. To address this issue, we analyzed two sets of 24-h dietary data and one dietary review dataset for sensitivity analysis, increasing the reliability of our conclusions. Another limitation is that the data were cross-sectional, meaning that we could not assess the long-term negative effects resulting from the interaction between OBS and PFASs. Additionally, there may be self-report bias, as some of the covariate data, such as liver disease, were self-reported. Finally, relying on a single blood sample to measure PFAS levels could introduce bias.

## 5. Conclusions

In conclusion, this study clarified the associations between OBS and PFOS, PFOA, and total PFASs and highlighted the importance of considering multiple aspects of dietary factors in future studies. This association was mainly significant for diet-related OBS. Both PFAS and OBS influence liver-related indicators in the blood. The effect of OBS on liver-related markers was greater in special PFASs level.

## Abbreviations

AA: Associate of Arts
ALP: Alkaline Phosphatase
ALT: Alanine Aminotransferase
AST: Aspartate Aminotransferase
BMI: Body Mass Index
CDC: Centers for Disease Control and Prevention
CI: Confidence Interval
DII: Dietary Inflammatory Index
GGT: Gamma-Glutamyl Transferase
LOD: Limit of Detection
MEC: Mobile Examination Center
MET: Metabolic Equivalent of Task
MUFA: Monounsaturated Fatty Acids
NCHS: National Center for Health Statistics
NHANES: National Health and Nutrition Examination Survey
OBS: Oxidative Balance Score
PFAS: Per- and Poly-fluoroalkyl Substances
PFDA: Perfluorodecanoic Acid
PFHxS: Perfluorohexane Sulfonic Acid
PFNA: Perfluorononanoic Acid
PFOA: Perfluorooctanoic Acid
PFOS: Perfluorooctane Sulfonic Acid
PIR: Poverty Income Ratio
PUFA: Polyunsaturated Fatty Acids
SFA: Saturated Fatty Acids
TB: Total Bilirubin
USDA: United States Department of Agriculture

## Supplementary information

**Additional file 1:** **Table S1:** Weighted characteristics of PFAS and OBS in participants. **Table S2:** Association between OBS and liver function markers after adjusted for PFAS level. **Table S3.** Association between PFAS and liver markers. **Figure S1.** Forest plots show the relationship between dietary-OBS and TB, ALP, GGT, ALT, and AST at different PFAS levels. **Figure S2.** Forest plots show the relationship between lifestyle-OBS and TB, ALP, GGT, ALT, and AST at different PFAS levels.

## Declarations

### Availability of data and materials

The data supporting the findings of this study are available at the NHANES website (https://www.cdc.gov/nchs/nhanes/index.htm) and can also be obtained from the corresponding author upon reasonable request.
