# Supplementary material for: Interaction between the oxidative balance score and serum per- and poly-fluoroalkyl substances (PFASs) on liver health: analysis of the NHANES 2007–2018 dataset
Source: Environ Health Prev Med. 2024 Oct 1;29:51. doi: 10.1265/ehpm.24-00159 (PMC11446636; doi:10.1265/ehpm.24-00159)

| **Table S1: Weighted characteristics of PFAS and OBS in participants** | | | | | | |  |
| --- | --- | --- | --- | --- | --- | --- | --- |
|  |  |  |  |  |  |  |  |
|  | Tertile 3 | Tertile 2 | | Tertile 1 | | Overall  P value |  |
|  |  | Value | P value compared T1 | Value | P value compared T1 |  |  |
| **PFOA, %** |  |  | 0.777 |  | 0.37 | 0.688 |  |
| T1 | 34.1 | 34.2 |  | 31.9 |  |  |  |
| T2 | 31.7 | 33.2 |  | 35.1 |  |  |  |
| T3 | 34.1 | 32.6 |  | 33 |  |  |  |
| **PFNA, %** |  |  | 0.201 |  | 0.152 | 0.261 |  |
| T1 | 37.2 | 33.8 |  | 35.4 |  |  |  |
| T2 | 29.2 | 33 |  | 34.1 |  |  |  |
| T3 | 33.6 | 33.2 |  | 30.5 |  |  |  |
| **PFOS, %** |  |  | 0.582 |  | 0.031 | 0.084 |  |
| T1 | 34 | 31.7 |  | 34.7 |  |  |  |
| T2 | 31.1 | 33.5 |  | 36.2 |  |  |  |
| T3 | 34.9 | 34.9 |  | 29.1 |  |  |  |
| **PFDA, %** |  |  | 0.574 |  | 0.13 | 0.235 |  |
| T1 | 35.6 | 32.9 |  | 33 |  |  |  |
| T2 | 39.2 | 40.6 |  | 44.5 |  |  |  |
| T3 | 25.3 | 26.5 |  | 22.5 |  |  |  |
| **PFHxS, %** |  |  | 0.948 |  | 0.683 | 0.91 |  |
| T1 | 34.3 | 34.5 |  | 32.4 |  |  |  |
| T2 | 32.1 | 32.7 |  | 34 |  |  |  |
| T3 | 33.6 | 32.7 |  | 33.5 |  |  |  |
| **Sum-PFAS, %** |  |  | 0.367 |  | 0.039 | 0.216 |  |
| T1 | 34.3 | 32.6 |  | 33.1 |  |  |  |
| T2 | 30.3 | 33.9 |  | 35.9 |  |  |  |
| T3 | 35.4 | 33.5 |  | 31.1 |  |  |  |
| Data are presented as weighted proportions. For comparisons between the two groups. Abbreviations: PFAS, perfluoroalkyl and polyfluoroalkyl substances; PFOA, perfluorooctanoic acid; PFNA, perfluorononanoic acid; PFOS, perfluorooctane sulfonic acid; PFDA, perfluorodecanoic acid; PFHxS, perfluorohexane sulfonic acid; OBS, oxidative balance score. | | | | | | |  |

| **Table S2:Association between OBS and liver function markers after adjusted for PFAS level.** | | | | |
| --- | --- | --- | --- | --- |
| Chemicals | OBS level | OBS | Dietary-OBS | Lifestyle-OBS |
|  |  | β (95%CI) | β (95%CI) | β (95%CI) |
| TBIL | High OBS | Reference | Reference | Reference |
|  | Moderate OBS | -0.015  (-0.081, 0.051) | -0.001  (-0.063, 0.061) | -0.007  (-0.071, 0.057) |
|  | Low OBS | -0.031  (-0.102, 0.039) | -0.015  (-0.093, 0.064) | 0.051  (-0.032, 0.134) |
|  | Per 1 unit OBS increase^*^ | 0.002  (-0.002, 0.006) | 0.002  (-0.002, 0.007) | -0.01  (-0.040, 0.020) |
|  | P trend^**^ | 0.579 | 0.681 | 0.311 |
| ALP | High OBS | Reference | Reference | Reference |
|  | Moderate OBS | 0.038  (0.000, 0.075) | 0.019  (-0.017, 0.055) | 0.035  (-0.005, 0.076) |
|  | Low OBS | 0.067  (0.022, 0.112) | **0.067**  **(0.027, 0.106)** | 0.023  (-0.04, 0.087) |
|  | Per 1 unit OBS increase^*^ | **-0.005**  **(-0.007, -0.002)** | **-0.005**  **(-0.008, -0.002)** | 0.009  (-0.007, 0.024) |
|  | P trend^**^ | 0.080 | **< 0.001** | 0.362 |
| GGT | High OBS | Reference | Reference | Reference |
|  | Moderate OBS | 0.012  (-0.054, 0.078) | -0.004  (-0.064, 0.057) | -0.086  (-0.175, 0.003) |
|  | Low OBS | 0.013  (-0.065, 0.092) | 0.002  (-0.078, 0.082) | **-0.178**  **(-0.302, -0.055)** |
|  | Per 1 unit OBS increase^*^ | -0.002  (-0.006, 0.003) | 0.000  (-0.005, 0.005) | **-0.038**  **(-0.066, -0.010)** |
|  | P trend^**^ | 0.974 | 0.94 | **0.007** |
| ALT | High OBS | Reference | Reference | Reference |
|  | Moderate OBS | -0.025  (-0.075, 0.024) | -0.023  (-0.070, 0.024) | -0.02  (-0.084, 0.044) |
|  | Low OBS | -0.059  (-0.127, 0.008) | -0.06  (-0.129, 0.008) | -0.014  (-0.102, 0.074) |
|  | Per 1 unit OBS increase^*^ | **0.005**  **(0.001, 0.009)** | **0.006**  **(0.002, 0.009)** | -0.016  (-0.036, 0.003) |
|  | P trend^**^ | **0.178** | **0.075** | 0.700 |
| AST | High OBS | Reference | Reference | Reference |
|  | Moderate OBS | -0.025  (-0.066, 0.017) | -0.015  (-0.052, 0.021) | 0.007  (-0.031, 0.045) |
|  | Low OBS | -0.056  (-0.105, -0.008) | -0.051  (-0.095, -0.006) | 0.062  (-0.009, 0.133) |
|  | Per 1 unit OBS increase^*^ | **0.004**  **(0.002, 0.007)** | **0.004**  **(0.001, 0.007)** | 0.01  (-0.003, 0.024) |
|  | P trend^**^ | **0.051** | **0.019** | 0.104 |
| Models were adjusted for survey cycle, sex, age, race/ethnicity, education level, smoking status, BMI, serum cotinine, PIR, alcohol consumption, and Sum-PFAS level. *: Calculated by including the OBS score directly as a continuous variable in the models. **: Calculated by entering the median value of each OBS category as a continuous variable in the models. Abbreviations: PFAS, perfluoroalkyl and polyfluoroalkyl substances; OBS, oxidative balance score; BMI, body mass index; PIR, poverty income ratio; CI, confidence interval. | | | | |

| **Table S2. Assocation between PFAS and liver markers** | | | | | | |
| --- | --- | --- | --- | --- | --- | --- |
|  | PFOA | PFNA | PFDA | PFOS | PFHxS | Sum-PFAS |
| TB | 0.043  (-0.003, 0.088) | 0.033  (-0.005, 0.071) | 0.006  (-0.036, 0.048) | 0.028  (-0.001, 0.056) | **0.037**  **(0.015, 0.06)** | **0.043**  **(0.009, 0.077)** |
| ALP | 0.010  (-0.013, 0.033) | -0.003  (-0.027, 0.021) | -0.003  (-0.023, 0.017) | 0.005  (-0.017, 0.028) | -0.004  (-0.019, 0.011) | 0.003  (-0.021, 0.027) |
| GGT | **0.040**  **(0.009, 0.071)** | 0.028  (-0.004, 0.061) | 0.030  (-0.001, 0.062) | -0.004  (-0.033, 0.024) | 0.004  (-0.021, 0.029) | 0.007  (-0.025, 0.039) |
| ALT | **0.031**  **(0.002, 0.061)** | **0.029**  **(0.003, 0.056)** | 0.001  (-0.025, 0.026) | 0.008  (-0.016, 0.031) | **0.034**  **(0.012, 0.055)** | 0.021  (-0.005, 0.047) |
| AST | **0.026**  **(0.007, 0.045)** | 0.015  (-0.009, 0.039) | -0.019  (-0.042, 0.004) | 0.000  (-0.017, 0.018) | **0.026**  **(0.010, 0.042)** | 0.011  (-0.008, 0.03) |
| Data are shown as β (95% CI), Bold indicates a P value less than 0.05. .Model were adjusted for survey cycle, sex, age, race/ethnicity, education level, smoking status, BMI, serum cotinine, PIR, energy intake, physical activity, have any liver disease, oxidative balance score, and alcohol consumption.  Abbreviations: CI, confidence interval; PFAS, perfluoroalkyl and polyfluoroalkyl substance; PFOA, perfluorooctanoic acid; PFNA, per-fluorononanoic acid; PFOS, perfluorooctane sulfonic acid, PFDA, perfluorodecanoic acid; PFHxS, perfluorohexanesulfonic acid; OBS, oxidative balance score; TB, total bilirubin; ALP, alkaline phosphatase; GGT, gamma-glutamyl transferase; AST, aspartate aminotransferase; ALT, alanine aminotransferase; BMI, body mass index; PIR, poverty income ratio. | | | | | | |

Figure S1. Forest plots show the relationship between Dietary-OBS and TB, ALP, GGT, ALT, and AST at different PFAS levels. Forest plots shown the regression coefficients (with 95% confidence intervals) between Dietary-OBS (as continuous variables) on liver markers across three different PFAS tertiles. The PFAS tertiles are defined as follows: Low PFAS (0-33%), Moderate PFAS (33%-66%), and High PFAS (66%-100%). Each square point on the plot represents the regression coefficient (β value), with error bars indicating the 95% confidence interval. The vertical line at 0 indicates no correlation. The linear regression was adjusted for the survey cycle, sex, age, race/ethnicity, education level, smoking status, BMI, serum cotinine, PIR, total energy intake, physical activity, history of liver disease, and alcohol consumption. Abbreviations: CI, confidence interval; PFAS, perfluoroalkyl and polyfluoroalkyl substance; PFOA, perfluorooctanoic acid; PFNA, per-fluorononanoic acid; PFOS, perfluorooctane sulfonic acid, PFDA, perfluorodecanoic acid; PFHxS, perfluorohexanesulfonic acid; OBS, oxidative balance score; TB, total bilirubin; ALP, alkaline phosphatase; GGT, gamma-glutamyl transferase; AST, aspartate aminotransferase; ALT, alanine aminotransferase; BMI, body mass index; PIR, poverty income ratio.


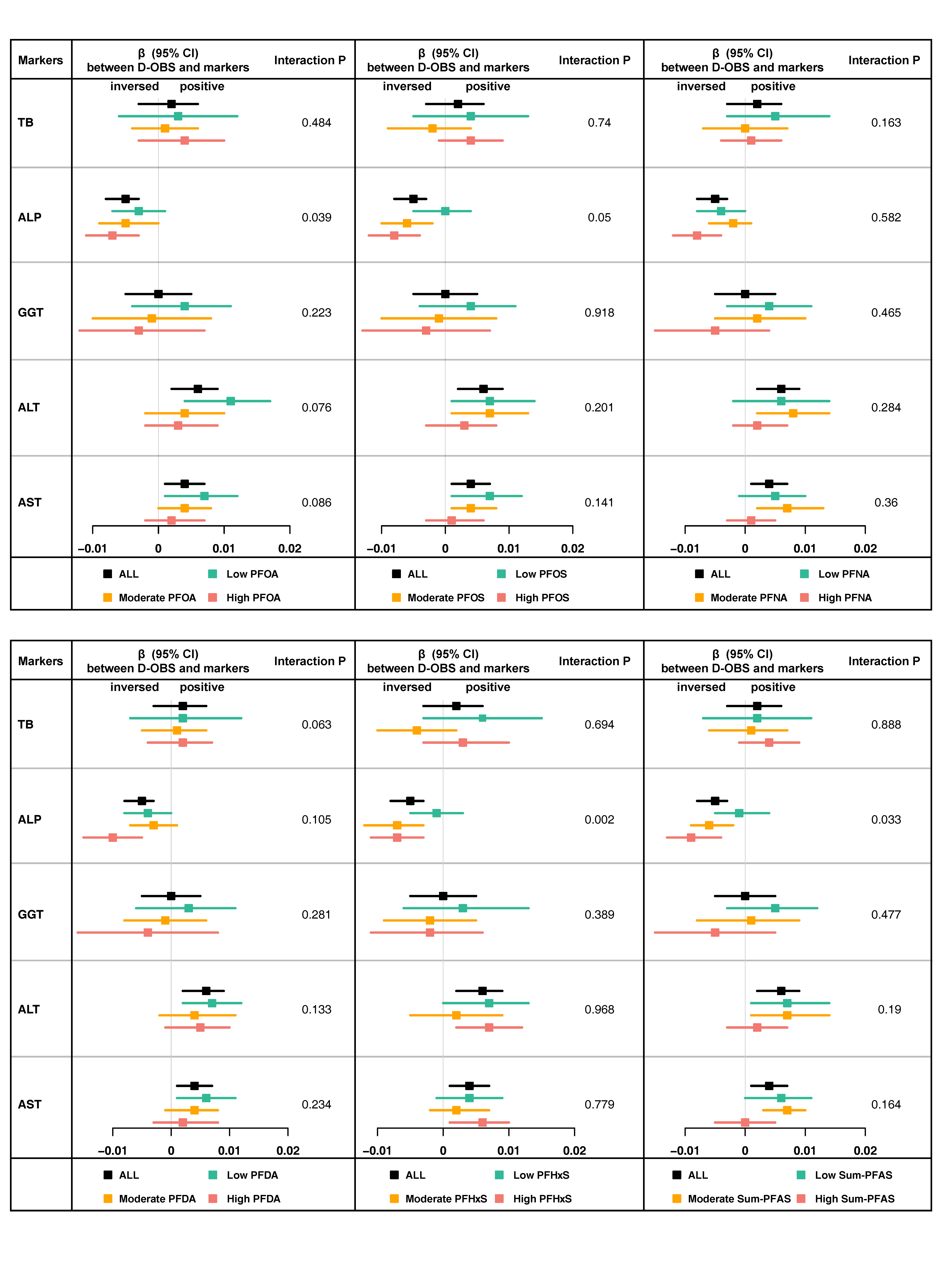


Figure S2. Forest plots show the relationship between Lifestyle-OBS and TB, ALP, GGT, ALT, and AST at different PFAS levels. Forest plots shown the regression coefficients (with 95% confidence intervals) between Lifestyle-OBS (as continuous variables) on liver markers across three different PFAS tertiles. The PFAS ter-tiles are defined as follows: Low PFAS (0-33%), Moderate PFAS (33%-66%), and High PFAS (66%-100%). Each square point on the plot represents the regression coefficient (β value), with error bars indicating the 95% confidence interval. The vertical line at 0 indicates no correlation. The linear regression was adjusted for the survey cycle, sex, age, race/ethnicity, education level, smoking status, BMI, serum cotinine, PIR, total energy intake, physical activity, history of liver disease, and alcohol consumption. Abbreviations: CI, confidence interval; PFAS, perfluoroalkyl and polyfluoroalkyl substance; PFOA, perfluorooctanoic acid; PFNA, per-fluorononanoic acid; PFOS, perfluorooctane sulfonic acid, PFDA, perfluorodecanoic acid; PFHxS, perfluorohexanesulfonic acid; OBS, oxidative balance score; TB, total bilirubin; ALP, alkaline phosphatase; GGT, gamma-glutamyl transferase; AST, aspartate aminotransferase; ALT, alanine aminotransferase; BMI, body mass index; PIR, poverty income ratio.


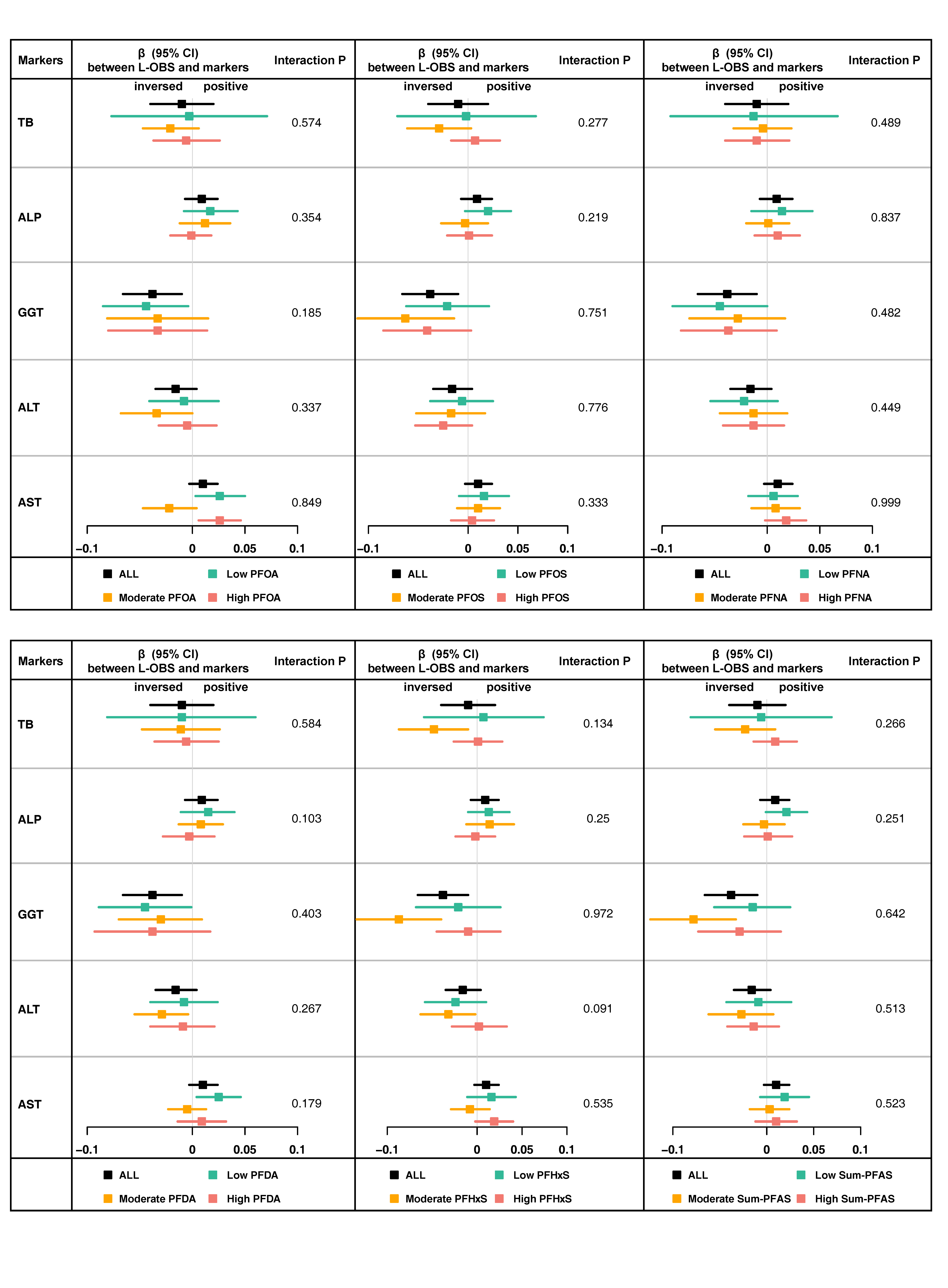

Supplement: Supplementary file 1 — Additional file 1: Table S1: Weighted characteristics of PFAS and OBS in participants. Table S2: Association between OBS and liver function markers after adjusted for PFAS level. Table S3. Association between PFAS and liver markers. Figure S1. Forest plots show the relationship between dietary-OBS and TB, ALP, GGT, ALT, and AST at different PFAS levels. Figure S2. Forest plots show the relationship between lifestyle-OBS and TB, ALP, GGT, ALT, and AST at different PFAS levels. [file ehpm-29-051-s001.docx]
